# Griffing's Methods Comparison for General and Specific Combining Ability in Cucumber

**DOI:** 10.1100/2012/524873

**Published:** 2012-04-19

**Authors:** J. A. Olfati, H. Samizadeh, B. Rabiei, Gh. Peyvast

**Affiliations:** ^1^Department of Horticultural Science, University of Guilan, Rasht 13488-1314, Iran; ^2^Department of Agronomy, University of Guilan, Rasht 13488-1314, Iran

## Abstract

A comparison among two forms of half-diallel analysis was made. The different half-diallel techniques used were Griffing's model I, method 2 and 4. These methods of diallel analysis were found to be interrelated. However, as Griffing's model I, method 4 partitioned heterosis into different components as well as gave information about combining ability and this method had certainly some advantages over the other. The results further indicated using parental generations in the second Griffing method may cause biased estimate of the GCA and SCA variances. Thus, using the fourth Griffing method is more suitable than the other methods in providing time, cost, and facilities, and it is recommended as an applicable method.

## 1. Introduction

Estimates of combining ability are useful in determining the breeding value of cucumber lines by suggesting the appropriate use in a breeding program. In studying combining ability, the most commonly utilized experimental approach is the diallel design. In the diallel analysis, Sprague and Tatum [1] introduced the concepts of general combining ability (GCA) and specific combining ability (SCA). The GCA is a measure of the additive genic action, while the SCA is assumed to be a deviation from additivity. Crossing a line to several others provides the mean performance of the line in all its crosses. This mean performance, when expressed as a deviation from the mean of all crosses, is called the general combining ability of the line. Any particular cross, then, has an expected value which is the sum of the general combining abilities of its two parental lines. The cross may, however, deviate from this expected value to a greater or lesser extent. This deviation is called the specific combining ability of the two lines in combination. In statistical terms, the general combining abilities are main effects and the specific combining ability is an interaction.

Griffing [2] defines diallel crosses in terms of genotypic values where the sum of general combining abilities for the two gametes is the breeding value of the cross (*i*, *j*). Similarly, specific combining ability represents the dominance deviation value in the simplest case ignoring epistatic deviation; see Kempthorne [3] and Mayo [4] for details.

Complete diallel cross designs involve equal numbers of occurrences of each of the distinct crosses among *p* inbred lines. Gupta and Kageyama [5], Dey and Midha [6], and Das et al. [7] investigated the issue of optimality of complete diallel crosses. When *p*, is large, or reciprocal crosses are similar to direct crosses it becomes impractical to carry out an experiment using a complete diallel cross design. In such situations, we use partial diallel cross designs where a subset of crosses are used. Although efficient designing of partial diallel crosses has been studied by several authors [8–11], no formal optimality result within adequately general classes has been reported except for the recent works of Mukerjee [12] and Das et al. [13]. Sometimes partial diallel crosses can, themselves, be quite large and thus it is desirable to use a block design for the experiment. Gupta et al. [14] and Mukerjee [12] provide orthogonal blocking schemes for partial diallel cross designs.

In the present paper, a comparative view of Griffing's model I, method 2 and 4 has been presented and discussed in light of their practical significance.

## 2. Matherial and Methods

To start with, 6 × 6 half diallel crosses of cucumber *(Cucumis sativus* L.) were produced. The varieties used were (1) “BH-502”, (2) “BH-504”, (3) “BH-604”, (4) “BH-605”, (5) “08wvc c-115”, (6) “08wvc c-118.” These crosses, along with their parents, were evaluated in a randomized block design with three replications. The following characteristics were recorded: early, unmarketable, marketable, and total yield; simple weight index (SWI). Simple weight index was calculated following Wehner and Cramer [15]. The data were analysed using the following models.

*Griffing's model I*

(i) Method 2: *Xij* = *u* + *gi* + *gj* + *sij* + (1/*b*)∑_*k*_ *e*_*ijk*_,
(ii) Method 4: *Xij* = *u* + *gi* + *gj* + *sij* + (1/*b*)∑_*k*_ *e*_*ijk*_
(*i* = *j* = 1 … *p*; *k* = 1 … *b*), where *u* = the population mean; *gi* = the general combining ability effect of the *i*th parent; *gj* = the general combining ability effect of the *j*th parent; *Sij* = the specific combining ability effect of the cross between *i*th and *j*th parents such that *slj* = *sji*; *e* *ijk* = the environmental effect associated with *ijk* th observation.

## 3. Results and Discussions

The analysis of variance for all measured traits carried out for testing the significance of genotypic differences is given in Table 1. The genotypic variance was also partitioned into its appropriate orthogonal components, namely, parents versus hybrids (Table 2). The genotypic differences were found significant. Significant differences were observed among the parents and hybrids. However, the significant differences of mean square associated with parents versus hybrids indicated availability of average heterosis for all traits. In Griffing's method 2, the variances due to gca and sca effects were highly significant for all traits (Table 3). However, the variance of early yield due to gca affects was not significant in method 4. On the other hand, the variance of early and nonmarketable yield due to sca effects was not significant (Table 4). The baker ratio in method 2 indicated the predominant role of additive type of gene effects for early yield, nonmarketable yield, and total yield while in method 4, this ratio indicated the predominant role of additive type of gene effects only for nonmarketable yield.

Heterosis tables showed that there are high heterosis for traits that show high SCA in method 4 Griffings (marketable yield, total yield, SWI, marketable yield percentage). In fact, this result indicated that method 4 is more suitable than method 2. Some authors believe that when the differences between hybrids and parents are significant, method 4 without parents entering in estimations is better that method 2 [16]. They in comparison of the second and fourth Griffing methods showed that the proportions of additive and nonadditive variances in two methods were different. Therefore, it could be concluded that using parental generations in the second Griffing method may cause biased estimate of the GCA and SCA variances [2]. Thus, using the fourth Griffing method is more suitable than the other methods in providing time, cost, and facilities, and it is recommended as an applicable method (Tables 5, 6, and 7).

## Figures and Tables

**Table 1:** ANOVA table effect of genotype on yield and some yield components.

Source of variation	Degree of freedom	Mean of square					
		Early yield	Marketable yield	Nonmarketable yield	Total yield	SWI	Marketable yield percentage
Block	2	0.006 ns	0.03 ns	0.006 ns	0.05 ns	0.002 ns	148.13 ns
Genotype	20	0.06**	1.31**	0.01**	1.39**	1.52**	441.26**
Error	40	0.01	0.07	0.003	0.06	0.08	107.84

C.V. (%)		10.71	12.73	10.51	11.15	6.51	11.91

ns, ** non significant and significant at *P* ≤ 0.01 respectively.

**Table 2:** Parent versus hybrids orthogonal comparisons.

	Early yield	Marketable yield	Nonmarketable yield	Total yield	SWI	Marketable yield percentage
Parents	1.13	1.60	0.51	1.68	4.61	93.46
Hybrids	1.03	2.31	0.58	2.49	4.37	84.65

Orthogonal test	4.71**	18.25**	3.76**	21.9**	18.21**	4.09**

**significant at *P* ≤ 0.01.

**Table 3:** Mean squares from diallel analysis for various characters in cucumber (Griffing's model I Method 2).

Source of variation	Degree of freedom	Mean of square					
		Early yield	Marketable yield	Nonmarketable yield	Total yield	SWI	Marketable yield percentage
GCA	5	0.13**	1.15**	0.026**	1.580**	1.37**	826.31**
SCA	15	0.036**	1.37**	0.007**	1.334**	1.57**	340.24**
M′e	40	0.004	0.02	0.001	0.021	0.03	35.95

MS_GCA_/MS_SCA_	—	3.61*	0.84 ns	3.71*	1.18 ns	0.87 ns	2.43 ns
Baker ratio	—	0.878	0.63	0.881	0.703	0.64	0.33
*h* ^2^_*n*_	—	0.35	b	0.31	0.04	b	0.23

ns, *, ** non significant and significant at *P* ≤ 0.05 and *P* ≤ 0.01 respectively.

b: not estimated because MS_GCA_ < MS_SCA_.

**Table 4:** Mean squares from diallel analysis for various characters in cucumber (Griffing's model I Method 4).

Source of variation	Degree of freedom	Mean of square					
		Early yield	Marketable yield	Nonmarketable yield	Total yield	SWI	Marketable yield percentage
GCA	5	0.01 ns	0.96**	0.016**	1.19**	0.16**	216.54**
SCA	9	0.01 ns	1.40**	0.002 ns	1.16**	1.57**	393.06**
M′e	28	0.003	0.03	0.001	0.02	0.04	29.08

MS_GCA_/MS_SCA_	—	1.00 ns	0.69 ns	8.00**	1.03 ns	0.10 ns	0.55 ns
Baker ratio	—	0.67	0.58	0.94	0.67	0.17	0.52
*h* ^2^_*n*_	—	b	b	0.64	b	b	b

ns, *, ** non significant and significant at *P* ≤ 0.05 and *P* ≤ 0.01 respectively.

b: not estimated because MS_GCA_ < MS_SCA_.

**Table 5:** High parent heterosis and mid parent heterosis for early yield and marketable yield.

Female Parent	Male parent	Early yield		Marketable yield	
		Mid parent heterosis	High parent heterosis	Mid parent heterosis	High parent heterosis
604	605	0.00	0.00	0.28	0.08
604	504	0.00	0.00	0.89	0.61
604	118	0.00	0.00	0.53	0.47
604	502	−0.07	−0.14	−0.06	−0.43
604	115	−0.31	−0.62	1.33	1.25
605	504	0.00	0.00	0.50	0.01
605	118	0.00	0.00	0.73	0.47
605	502	−0.07	−0.14	0.42	−0.15
605	115	−0.31	−0.62	1.53	1.24
504	118	0.00	0.00	1.30	1.07
504	502	−0.07	−0.14	−0.16	−0.24
504	115	−0.17	−0.49	1.36	1.16
118	502	0.07	0.00	1.00	0.69
118	115	−0.31	−0.62	−0.41	−0.44
502	115	−0.24	−0.49	1.30	1.02

**Table 6:** High parent heterosis and mid parent heterosis for nonmarketable yield and total yield.

Female Parent	Male parent	Nonmarketable yield		Total yield	
		Mid parent heterosis	High parent heterosis	Mid parent heterosis	High parent heterosis
604	605	0.04	0.04	0.418	0.213
604	504	0.12	0.12	1.077	0.793
604	118	0.08	0.08	0.697	0.643
604	502	0.12	0.12	0.215	−0.150
604	115	0.08	0.00	1.330	1.020
605	504	0.08	0.08	0.672	0.183
605	118	0.04	0.04	0.822	0.563
605	502	0.04	0.04	0.510	−0.060
605	115	0.11	0.03	1.588	1.073
504	118	0.04	0.04	1.357	1.127
504	502	0.11	0.11	0.068	−0.013
504	115	0.08	0.00	1.337	1.310
118	502	0.07	0.07	1.115	0.803
118	115	0.04	−0.03	−0.257	−0.513
502	115	0.08	0.00	1.278	1.223

**Table 7:** High parent heterosis and mid parent heterosis for SWI and marketable yield percentage.

Female Parent	Male parent	SWI		Marketable yield percentage	
		Mid parent heterosis	High parent heterosis	Mid parent heterosis	High parent heterosis
604	605	−0.72	−0.77	0.00	0.00
604	504	−0.90	−1.00	−13.10	−13.10
604	118	−0.55	−0.57	−15.00	−15.00
604	502	−1.36	−1.50	−26.11	−26.11
604	115	0.91	−0.25	5.46	−14.17
605	504	−0.78	−0.93	−15.00	−15.00
605	118	−0.30	−0.37	−8.33	−8.33
605	502	−0.42	−0.61	−8.33	−8.33
605	115	0.82	−0.29	1.90	−17.73
504	118	0.49	0.41	−3.03	−3.03
504	502	−1.53	−1.57	−17.86	−17.86
504	115	0.87	−0.39	7.99	−11.64
118	502	−0.62	−0.75	−7.41	−7.41
118	115	−0.71	−1.89	−24.81	−44.44
502	115	1.13	−0.16	8.14	−11.49
